# Neuropathology consultation rates in medico-legal autopsies show substantial within-country variation—a nationwide Finnish study

**DOI:** 10.1093/fsr/owad027

**Published:** 2023-10-10

**Authors:** Petteri Oura, Aki Eklin, Antti Sajantila

**Affiliations:** Department of Forensic Medicine, Faculty of Medicine, University of Helsinki, FI-00014, Helsinki, Finland; Forensic Medicine Unit, Finnish Institute for Health and Welfare, FI-00271, Helsinki, Finland; Forensic Medicine Unit, Finnish Institute for Health and Welfare, FI-00271, Helsinki, Finland; Department of Forensic Medicine, Faculty of Medicine, University of Helsinki, FI-00014, Helsinki, Finland; Forensic Medicine Unit, Finnish Institute for Health and Welfare, FI-00271, Helsinki, Finland

**Keywords:** neuropathology, consultation, forensic pathology, systems research, Finland

## Abstract

Neuropathology consultations are an essential part of medico-legal cause-of-death investigations. However, there are little data on the rates of neuropathological examinations in medico-legal autopsies. The present nationwide, retrospective, register-based study aimed to report and compare neuropathology consultation rates (i.e. the percentages of medico-legal autopsies with a neuropathology consultation) in five Finnish regions from 2016 to 2021. The dataset comprised 50 457 medico-legal autopsies with 1 274 neuropathology consultations. Overall, ~1 in 40 autopsies (2.5%) involved a neuropathology consultation. Consultation rates were lowest in the Southern Finland region (1.4%) and highest in the Southwestern Finland and Åland region (6.5%). Throughout the study period, the consultation rates of Southwestern Finland and Åland were 1.5 to 9.4 times those of other regions (*P* < 0.001). In conclusion, this nationwide Finnish study identified substantial differences in neuropathology consultation rates between regions, which may indicate regional differences in conventions and policies. However, the “optimal” consultation rate remains unknown. Future studies are required to further understand the differences in autopsy practices within the Finnish context as well as in medico-legal institutions elsewhere.

**Key points:**

- There are little data on the rates of neuropathology consultations in medico-legal autopsies.
- This Finnish study characterized regional differences in neuropathology consultation rates between 2016 and 2021.
- Overall, 1 in 40 autopsies (2.5%) involved a neuropathology consultation.
- The consultation rates of Southwestern Finland and Åland were 1.5 to 9.4 times those of other regions.
- Our findings may reflect regional differences in conventions and policies.

## Introduction

Pathological and traumatic changes in the central nervous system are often relevant to the cause of death; they are therefore of major interest in forensic pathology [1–3]. However, appropriate examination of the central nervous system often requires special expertise and complex processing techniques [2, 4–7], and the conclusions can play a pivotal role in legal proceedings [3, 8]. Given that this expertise may be outside the scope of general forensic pathologists, consultations with neuropathologists have become an integral part of high-quality cause-of-death investigation practices [1, 3, 8].

Despite scientific advancements in neuropathological methodology in recent years [9], data on the use of neuropathology in medico-legal autopsies remain lacking. A study from Macedonia [10] assessed a series of 80 cases with closed head injuries and concluded that neuropathological examination was the only way to establish the exact diagnosis of brain injury, which often constituted the cause of death. To further evaluate the importance of neuropathology consultations for medico-legal cause-of-death investigations, it is important to assess consultation frequency as well as inter-institutional variation in representative datasets. However, there is a paucity of reports on neuropathology consultation rates in medico-legal institutions.

In Finland, all medico-legal autopsies are performed in five regional offices of the Finnish Institute for Health and Welfare, which provides a solid basis for a nationwide analysis. The aim of this study was 2-fold: first, to report neuropathology consultation rates (i.e. the percentage of medico-legal autopsies with a neuropathology consultation) at a nationwide level, and second, to compare consultation rates between the five regional offices.

## Materials and methods

### Study protocol

This retrospective, register-based study used nationwide data on the number of medico-legal autopsies and associated neuropathology consultations performed in Finland between 2016 and 2021. All medico-legal autopsies that were performed within the timeframe (*n* = 50 457) [11] were included; there were no exclusions. Ethical approval was not required because the study was based on public aggregate-level data released by the Finnish Institute for Health and Welfare.

### Medico-legal autopsies and neuropathology consultations

A cause-of-death investigation is mandatory for all deaths that occur in Finland (Act on the Investigation of the Cause of Death 1973/459). The investigation is either performed as a medical examination that may involve a clinical autopsy, or as a police-led medico-legal investigation that usually involves a medico-legal autopsy [12]. A medico-legal investigation is required in cases of suspected homicide, suicide, accidental death, medical or surgical adverse events, or occupational diseases, as well as in cases of sudden or unexpected death. The medico-legal autopsy rate in Finland is generally high [13, 14], currently around 15%.

Nationally, the Finnish Institute for Health and Welfare is the sole authority in charge of medico-legal autopsies. The Forensic Medicine Unit has regional offices in five major university cities across Finland; each office is responsible for performing medico-legal autopsies in their region (Helsinki office: Southern Finland, Turku office: Southwestern Finland and Åland, Tampere office: Western and Inland Finland, Kuopio office: Eastern Finland, Oulu office: Northern Finland and Lapland). In 2016, the Forensic Medicine Unit established a nationwide electronic information system (OLT) for comprehensive medico-legal documentation. This information system includes records of all medico-legal autopsies and ancillary investigations performed nationally.

In this study, we queried the nationwide electronic information system for aggregate-level data on the total number of medico-legal autopsies and neuropathology consultations performed in each of the five regions from 2016 to 2021. The total number of cause-of-death investigations involving an autopsy was collected as the total number of autopsies. Neuropathology consultations were identified from electronic referrals to a specialist in neuropathology (e.g. a full neuropathological examination, a neuropathological examination of specific tissue samples, or a neuropathological assessment of microscope slides). However, it is important to note that neuropathologists have a strictly consultative role in cause-of-death investigations; the responsibility of determining the cause of death remains solely with the forensic pathologist.

### Statistical analysis

Total numbers of medico-legal autopsies and neuropathology consultations were first presented as raw counts. To adjust for regional differences in autopsy volumes, neuropathology consultation rates (i.e. the percentages of medico-legal autopsies with a neuropathology consultation) were then calculated. The data were presented individually for each year and region.

To perform statistical comparisons between regions, a generalized estimating equations model [15] was constructed, with neuropathology consultation rate as the outcome and region and year as predictor variables. Annual data were considered to be nested within regions.

SPSS version 27 (IBM Corp., Armonk, NY, USA) was used to perform the statistical analyses, and *P* = 0.05 was selected as the threshold for significance.

## Results

Nationally, the dataset comprised 50 457 medico-legal autopsies and 1 274 neuropathology consultations over the 6-year study period (Table 1). Overall, ~1 in 40 autopsies (2.5%) was associated with a neuropathology consultation. Consultation rates were lowest in Southern Finland (1.4%) and highest in Southwestern Finland and Åland (6.5%).

**Table 1:** Total numbers of medico-legal autopsies and neuropathology consultations presented by region and year.

Region	Year						
	2016	2017	2018	2019	2020	2021	2016–2021
Southern Finland							
Medico-legal autopsies (*n*)	3 028	3 121	3 233	3 075	3 218	3 162	18 837
Neuropathology consultations (*n*)	39	52	66	31	52	15	255
Neuropathology consultation rate (%)	1.3	1.7	2.0	1.0	1.6	0.5	1.4
Southwestern Finland and Åland							
Medico-legal autopsies (*n*)	1 445	1 254	1 104	1 132	1 043	1 071	7 049
Neuropathology consultations (*n*)	96	90	88	77	61	45	457
Neuropathology consultation rate (%)	6.6	7.2	8.0	6.8	5.8	4.2	6.5
Western and Inland Finland							
Medico-legal autopsies (*n*)	1 980	1 943	1 905	1 741	1 749	1 700	11 018
Neuropathology consultations (*n*)	39	59	51	69	38	9	265
Neuropathology consultation rate (%)	2.0	3.0	2.7	4.0	2.2	0.5	2.4
Eastern Finland							
Medico-legal autopsies (*n*)	1 272	1 214	1 231	1 145	1 121	1 170	7 153
Neuropathology consultations (*n*)	16	30	30	37	44	6	163
Neuropathology consultation rate (%)	1.3	2.5	2.4	3.2	3.9	0.5	2.3
Northern Finland and Lapland							
Medico-legal autopsies (*n*)	1 074	1 048	1 078	1 044	1 070	1 086	6 400
Neuropathology consultations (*n*)	8	28	32	29	26	11	134
Neuropathology consultation rate (%)	0.7	2.7	3.0	2.8	2.4	1.0	2.1
All regions pooled							
Medico-legal autopsies (*n*)	8 799	8 580	8 551	8 137	8 201	8 189	50 457
Neuropathology consultations (*n*)	198	259	267	243	221	86	1 274
Neuropathology consultation rate (%)	2.3	3.0	3.1	3.0	2.7	1.1	2.5

**Figure 1:**
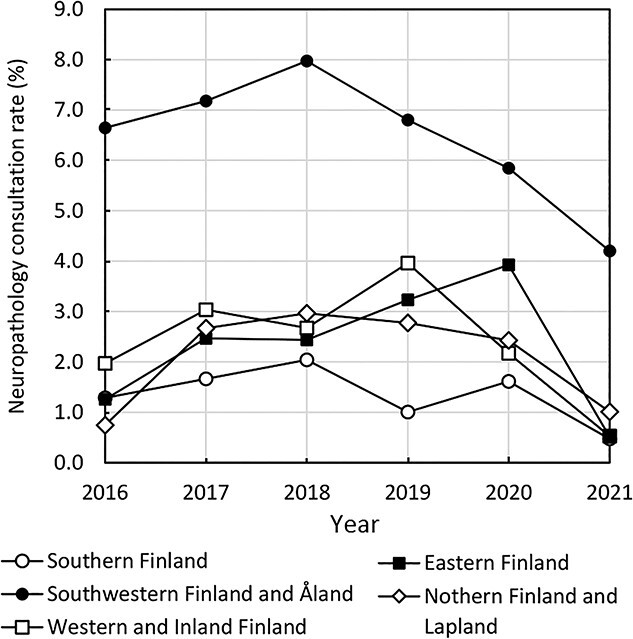
Neuropathology consultation rates (i.e. the percentages of medico-legal autopsies with a neuropathology consultation) presented by region and year.

Regional and temporal trends in neuropathology consultation rates are presented in Figure 1. The consultation rates of Southern Finland, Western and Inland Finland, Eastern Finland, and Northern Finland and Lapland showed some fluctuations but were of relatively similar magnitudes, ranging between 0.5% and 4.0%. Southwestern Finland and Åland had the highest consultation rates throughout the study period, ranging between 4.2% and 8.0%. These rates were 1.5 to 9.4 times those of the other regions (*P* < 0.001).

## Discussion

This Finnish study, which comprised over 50 000 medico-legal autopsies between 2016 and 2021, identified substantial regional differences in neuropathology consultation rates. Southwestern Finland and Åland had the highest consultation rates throughout the study period, ranging between 4.2% and 8.0%. These rates were 1.5 to 9.4 times those of other regions.

Approximately 1 in 40 autopsies (2.5%) was associated with a neuropathology consultation. Given that the medico-legal autopsy rate in Finland is relatively high, our findings indicate that neuropathology does not play a critical role in most cases. However, the selection of cases referred for medico-legal autopsy may vary significantly between countries and institutions; a lack of reports from other institutions impedes international and inter-institutional comparisons.

Although the importance of forensic neuropathology has previously been reported in the context of, for example, closed head injuries [10], there are virtually no data addressing the “optimal” neuropathology consultation rate for medico-legal systems. We acknowledge that optimal rates are heavily influenced by national legislation, local cause-of-death investigation practices, resources, population characteristics, and autopsy volume. In our study, we did not aim to assess the additional benefits provided by a neuropathological examination, and were therefore unable to address the optimal consultation rate in the Finnish medico-legal system. However, the future establishment of an optimal consultation rate may allow for a cost-effectiveness analysis at a centre- or system-specific level.

In region-specific scrutiny, our analysis revealed substantial differences in neuropathology consultation rates between offices. Consultation rates were lowest in Southern Finland (1.4%) and highest in Southwestern Finland and Åland (6.5%). In particular, the Southwestern Finland and Åland region stood out because it had neuropathology consultation rates that were 1.5 to 9.4 times those of any other region throughout the study period. We speculate that this finding may indicate regional differences in conventions, perspectives, and/or policies. Future studies are required to evaluate at least two aspects. First, to investigate whether similar regional differences exist within medico-legal systems and units outside of Finland; we hypothesize that the observed phenomenon may be globally prevalent. Second, to explore the factors underlying regional discrepancies within the Finnish medico-legal system.

The theoretical framework of health service utilization emphasizes societal, system-related, and individual characteristics (e.g. technology, policy, population, resources, availability, cooperation, system complexity, quality, and professionals) as major determinants of service utilization [16, 17]. From this viewpoint, education, professional activity, and know-how may also explain some of the regional variation in neuropathology consultation rates observed in this study. However, these components lie outside the boundaries of the present dataset.

The main strengths of this study include its nationwide approach, the high volume of medico-legal autopsies, and a relatively long study period (extending over 6 years). Data on autopsies and consultations were extracted from a reliable nationwide electronic information system. Moreover, regional variations in autopsy volumes were accounted for by calculating consultation rates, which were comparable across regions. Finally, because all offices operate under the same authority and perform autopsies based on the Act on the Investigation of the Cause of Death, between-region variability in case profiles is expected to be minor.

The main limitation of the present analysis was the lack of background data; for example, sex, age, and causes of death were unavailable. Furthermore, although the general trends were clear, the present dataset did not allow a detailed analysis of any underlying factors. Additionally, our use of aggregate-level data meant that we were unable to differentiate between full neuropathological examinations and other consultation types (e.g. regarding more specific samples or microscope slides). Future analyses should aim to compensate for these limitations.

Future perspectives are manifold. Primarily, studies should aim to report and compare neuropathology consultation rates in other medico-legal systems. The collection of essential background data will also be necessary to understand differences between systems. We believe that essential data include case demographics, the forensic pathologist’s reasons for consultation, tissue processing and staining methods, common findings, and the benefit of the neuropathologist’s report for the cause-of-death investigation. These aspects can likely be covered *via* a retrospective chart review. Finally, prospective approaches are required to establish uniform criteria and standardized autopsy protocols for cases with potential neuropathology relevance (e.g. suspected brain injury [4]).

In conclusion, this nationwide Finnish study identified substantial regional differences in neuropathology consultation rates. Throughout the study period, the Southwestern Finland and Åland region had rates that were 1.5 to 9.4 times those of any other region. This finding may indicate regional differences in conventions, perspectives, and policies. However, the optimal consultation rate remains unknown. Future studies are required to better understand the differences in autopsy practices within the Finnish context, as well as in medico-legal institutions elsewhere.

## Authors' contributions

Petteri Oura conceived of the study, participated in its design and coordination, performed the data analysis, and drafted the manuscript. Aki Eklin participated in data collection and helped to draft the manuscript. Antti Sajantila participated in the study’s design and coordination and helped to draft the manuscript. All authors contributed to the final text and approved it.

## Compliance with ethical standards

Ethical approval was not required because the study was based on public aggregate-level data released by the Finnish Institute for Health and Welfare.

## Disclosure statement

The authors report there are no competing interests to declare.

## Funding

No funding was received for this study.
